# Improving the Ambient Temperature Control Performance in Smart Homes and Buildings

**DOI:** 10.3390/s21020423

**Published:** 2021-01-09

**Authors:** Fernando Fontes, Rómulo Antão, Alexandre Mota, Paulo Pedreiras

**Affiliations:** 1 Departament of Electronics, Telecommunications and Informatics (DETI), University of Aveiro, 3810-193 Aveiro, Portugal; romuloantao@ua.pt (R.A.); alex@ua.pt (A.M.); pbrp@ua.pt (P.P.); 2 Instituto de Telecomunicações, Campus de Santiago, 3810-193 Aveiro, Portugal

**Keywords:** smart buildings temperature control, pol-placement, system identification, low-cost sensors

## Abstract

Currently, it is becoming increasingly common to find numerous electronic devices installed in office and residential spaces as part of building automation solutions. These devices provide a rich set of data related to the inside and outside environment, such as indoor and outdoor temperature, humidity, and solar radiation. However, commercial of-the-shelf climatic control systems continue to rely on simple controllers like proportional-integral-derivative or even on-off, which do not take into account such variables. This work evaluates the potential performance gains of adopting more advanced controllers, in this case based on pole-placement, enhanced with additional variables, namely solar radiation and external temperature, obtained with dedicated low-cost sensors. This approach is evaluated both in simulated and real-world environments. The obtained results show that pole-placement controllers clearly outperform on-off controllers and that the use of the additional variables in pole-placement controllers allows relevant performance gains in key parameters such as error signal MSE (17%) and control signal variance (40%), when compared with simple PP controllers. The observed energy consumption savings obtained by using the additional variables are marginal (≈1%, but the reduction of the error signal MSE and control signal variance have a significant impact on energy consumption peaks and on equipment lifetime, thus largely compensating the increase in the system complexity.

## 1. Introduction

Smart Homes and Buildings (SHaB) are becoming a reality, and in these spaces, it is increasingly common to find products with connectivity features and ecosystem integration [1]. The goals of smart homes and buildings are many-fold, including the improvement of the user’s quality of life, comfort, safety, and security, while bringing improved resource usage efficiency.

SHaB management systems can control several services, including lighting, air conditioning, entertainment systems, and appliances, to reduce the need for human interaction while improving energy efficiency and sustain the desired user comfort levels [2]. This goal is in route with the most recent initiatives towards the energy consumption reduction for air conditioning systems. As an indicative value, the burden of the energy taken by these systems in the USA is estimated to be around 20% of the total consumption [3].

Climate control systems are part of a set of technologies commonly referenced in the literature as “Heating, Ventilation, and Air Conditioning” (HVAC) that aim to provide thermal comfort and proper indoor air quality in closed spaces such as houses, industrial settings, commercial spaces, and cars. HVAC systems are expected to be autonomous and efficient energy-wise, providing adequate comfort levels and, when applicable, assuring compliance with the safety requirements. However, the spaces in which these systems are deployed are heterogeneous and, often, dynamic. Aspects such as the type and materials used in the construction, the number and surface of windows, solar exposure, the utilization of machinery, the number of users, and the kind of activities they perform, to name just a few, have a strong and direct influence on the thermal behavior of the spaces; thus, in practice, these systems may underperform and fail to meet the user requirements. This problem is acknowledged by air-conditioning manufacturers (e.g., Honeywell (https://www.honeywellhome.com/en/products/thermostat), Nest (https://nest.com/thermostats/nest-learning-thermostat/tech-specs/)), which in some cases have special operation modes that by-pass the normal control algorithm, e.g., “party mode” in which the pieces of equipment are set to the highest flow rate volume.

Since all these variables influence the control process, failing to take them into account weakens the performance of HVAC systems, including energy consumption (total amount, pattern, and peaks) and comfort. In fact, conventional temperature controllers only use the instantaneous interior house temperature, along with the desired temperature (setpoint). However, a more advanced controller that accounts for other data sources, such as the daily weather forecast, the incident solar radiation, and the external house temperature, may perform much more efficiently [4]. If such factors prove to have a significant impact, they may compensate the additional controller complexity and so become a viable option.

In a previous contribution [5], the authors evaluated the use of adaptive control strategies, namely pole-placement and model-based predictive control, as a way to improve the performance of temperature control systems in the face of the dynamic characteristics of buildings. This work builds upon that contribution, by enriching the controller learning process with additional external variables, the outside temperature in this case, and assessing the respective realization costs and performance gains. The proposed solution was evaluated both in simulation and in a physical installation. The facilities where the experiments were carried out did not possess devices that could provide the external temperature; therefore, a dedicated low-cost temperature sensor was deployed to acquire this variable.

The paper is organized as follows. Section 2 overviews some of the more relevant scientific contributions related to the topic addressed in this paper. Section 3 briefly presents the dynamic model of the building environment that was devised to account for the external environment conditions. Section 4 details the setup used to validate the proposed solution, including the instrumentation system used to carry out the measurements of complementary variables, such as solar radiation. Section 5 presents the controller design. Section 6 and Section 7 discuss simulation and real-world results, respectively. Finally, Section 8 presents the main conclusions of this work.

## 2. Related Work

Climate control systems are required to keep the environment temperature within a desired temperature profile. The literature reports two main categories of controllers, open-loop and closed-loop. An open-loop strategy can be deployed, e.g., by using a preset timer to activate or deactivate the heating system or by using a feed-forward control strategy based on an approximate model of the system [6]. These systems are extremely simple and inexpensive, as they have minimal processing and hardware requirements because they do not depend on real-time measurements of any environment variables, such as inside and outside temperature or solar radiation. However, such an approach can lead to a significant waste of energy and poor comfort levels, because the control system has no means to anticipate or even react to variations in the real scenarios. For example, the external temperature and solar radiation have significant fluctuations over the year, and the evolution profiles of these variables during the day also vary significantly. This is an inherent, well-known, and generic limitation of open-loop controllers. On the other hand, closed-loop controllers solve this problem by including a feedback path in which both the setpoint and sensor data are present, thus allowing measuring the error and taking appropriate actions, i.e., adapting the control signal, in order to drive the system close to the desired state. Closed-loop controllers are more complex and expensive than open-loop controllers, as they need to collect sensor data, process them, and execute a control algorithm, but their performance is much better, enabling a more efficient use of the available energy resources and increased comfort levels.

There is an extensive record of scientific contributions in the area of indoor temperature control systems, including closed-loop control architectures. The energy consumption of basic on-off controllers was evaluated in [7]. An extensive survey on closed-loop control algorithms for HVAC systems was presented in [3]. In [8], Proportional-Integral-Derivative (PID) controllers were considered, evaluating the impact of their parameters in the overall energy efficiency of HVAC systems. Following a polynomial controller synthesis approach and using a simulation environment for the analysis, Reference [9] proposed a temperature controller for a generic enclosed space based on robust control theory, analyzing the impact of the model parameter’s tolerances over the system stability analysis. On the other hand, in [10], the use of fuzzy logic controllers was proposed, and an optimization method for such architectures was presented. More advanced controllers have also been considered in the literature. For example, neural networks were considered in [11,12], where the use of Model Predictive Control (MPC) was proposed, while in [13,14], deep-learning based implementations were proposed.

Abundant references can also be found in areas closely related to building indoor temperature control, such as greenhouse environment control, which has requirements and challenges that, in some aspects, approach the application scenarios addressed in this paper. Fuzzy logic controllers have been tested for a long while now. These controllers have the benefit of allowing the specification of different profiles for different desired outcomes. In particular, while in the context of greenhouses, controllers aim at efficiency rather than comfort, it is possible to define sets of rules for distinct requirements and objectives, namely buildings. It is known that the major problems of fuzzy logic controllers are the training and the definition of the set of rules. In [15], a human expert was used for creating this set of rules. However, in both [16,17], the use of a genetic algorithm was proposed to solve this problem. The impact of the incident solar radiation, wind speed, and outside temperature was also discussed. In these references, the proposed methods were evaluated only by simulation, using MATLAB/Simulink. These works are complementary to our proposal since their focus was only on the optimization of the controller itself and not on the benefits of introducing the outdoor variables into the regression phase.

Merging the concepts of fuzzy logic and neural networks, Reference [18] presented an adaptive neuro-fuzzy controller (ANFIS) to adjust the internal temperature of a greenhouse, validating the method using a comprehensive dynamic environment model using the MATLAB/Simulink environment. In [19], the non-linear dynamics of the greenhouse environment was approached using Volterra series to develop a non-linear model predictive controller. The environment was modeled using high-order polynomial models, and the control action was obtained via a non-linear optimization problem, by iteratively solving a Quadratic Programming (QP) problem. The method was validated in a simulation environment, as well as in a real application scenario.

In order to overcome the computational complexity inherent to developing a control strategy based on non-linear models, Reference [20] proposed a model predictive control strategy based on feedback linearization, i.e., approximating the model with linear models valid over the current operation point, leading to an easier implementation of the MPC algorithm and a significant reduction of the computational burden involved in solving the non-linear optimization problem. The method was evaluated in a simulation environment using a discrete time model, extracted from several datasets obtained in the field.

On the other hand, Reference [21] proposed an alternative for solving the complex problems inherent to model predictive control. The authors proposed the use of the particle swarm optimization algorithm to overcome the design challenges when performing air temperature control. Simulation results compared this approach with the ones obtained by using genetic and sequential quadratic programming algorithms. The external variables like solar radiation were used in the regression vector, but no performance analysis was made.

In [22], the authors proposed a multirate adaptive temperature control of greenhouses to overcome the instability problems and the large diversity exhibited by such structures. Instead of feeding into the regression vector the external variables, this work proposed to use the idle time between the successive actuation periods to take multiple samples of the current indoor temperature for constantly re-estimating the parameters of the indoor model. Simulation results were presented for both the pole-placement and the linear quadratic regulation controllers.

This work is grounded in the hypothesis that the use of advanced and self-adaptive control strategies that take into account, in addition to the internal temperature, a richer set of data sources, including external conditions such as incident solar radiation and external house temperature, may outperform systems that rely solely on internal temperature. The literature review above allows us to conclude that in most of the cases, the external environment variables are not considered in the realization of the controllers. In the scenarios where they are, the non-linear nature of this control problem arises, and to take advantage of the additional sources of information for an optimized actuation decision, either iterative non-linear optimization methods are used or local linearizations are performed in order to implement a practical control system. The real-time model identification procedure that supports our control strategy overcomes this need by using a linear approximation of the controlled non-linear process that is continuously updated in real-time, enabling as well the use of advanced linear control strategies, which can be defined as a closed-form mathematical problem that provides a sub-optimal yet simpler solution for the closed loop reference tracking goal. Finally, in most of the cases, the evaluation of the approaches is carried out only by simulation, and practical realization aspects are disregarded.

## 3. Indoor Environment Model

Developing a thermodynamic model for an indoor environment is not a simple task. It is common to find models based on the energy balance equations of the interfaces (such as windows, floor, walls, and ceiling) that constitute the boundaries of the indoor environment (e.g., [23]). However, to be effective, this method requires an accurate determination of many parameters, like the thermal resistance coefficient of the surfaces, which are hard or even impossible to obtain. Alternative approaches, often adopted for the study of buildings’ energy efficiency, include the use of finite element analysis (e.g., [24,25,26]).

Since we are interested in studying the dependence on variable parameters, such as the incident solar radiation or the external environmental temperature, we assume that it is adequate to use a model based on elementary equations that includes the outdoor environment, the thermal characteristics of a standard house, and the heating system. The justification for choosing this simpler model is that the more elaborated models, mentioned above, are very complex and hard to simulate and implement, because they have several non-linearities, which have a significant impact in the loop gain, eventually leading to instabilities in the control algorithm. This problem is further aggravated by the fact that the parameters are dynamic. Therefore, to limit the complexity of the realization of the controller to an acceptable level, a dynamic model governed by system’s equations was adopted (1), adapted from [27].

(1) $$\begin{array}{cc} \; & \left(\frac{\partial T_{heater}}{\partial t}\right)=\left(\frac{T_{heater\_avg}}{\tau_{heater}}u-\frac{1}{\tau_{heater}}\cdot T_{heater}\right)\\ \; & {\left(\frac{\partial Q}{\partial t}\right)}_{heater}=\left(T_{heater}-T_{room}\right)\cdot M_{dot}\cdot c\\ \; & {\left(\frac{\partial Q}{\partial t}\right)}_{losses}=\frac{T_{room}-T_{out}}{R_{eq}}\\ \; & \left(\frac{\partial T_{room}}{\partial t}\right)=\frac{1}{M_{air}\cdot c}\cdot \left(\frac{\partial Q_{heater}}{\partial t}-\frac{\partial Q_{losses}}{\partial t}+S_{rad}\cdot W_{area}\right) \end{array}$$

where,

$$\begin{array}{c} \begin{array}{cc} \frac{\partial Q}{\partial t} & =\mathrm{heat}\;\mathrm{flow}\;\mathrm{from}\;\mathrm{the}\;\mathrm{heater}\;\mathrm{into}\;\mathrm{the}\;\mathrm{room}\;(W/m^{2})\\ c & =\mathrm{heat}\;\mathrm{capacity}\;\mathrm{of}\;\mathrm{air}\;\mathrm{at}\;\mathrm{constant}\;\mathrm{pressure}\;(J/(\mathrm{kg}\cdot K))\\ M_{dot} & =\mathrm{air}\;\mathrm{mass}\;\mathrm{flow}\;\mathrm{rate}\;\mathrm{through}\;\mathrm{the}\;\mathrm{heater}\;(\mathrm{kg}/s)\\ T_{heater} & =\mathrm{temperature}\;\mathrm{from}\;\mathrm{the}\;\mathrm{heater}\;{(}^{\circ }C)\\ T_{heater\_avg} & =\mathrm{average}\;\mathrm{heater}\;\mathrm{temperature}\;{(}^{\circ }C)\\ \tau_{heater} & =\mathrm{heater}\;\mathrm{time}\;\mathrm{constant}\;(s)\\ T_{room} & =\mathrm{current}\;\mathrm{room}\;\mathrm{air}\;\mathrm{temperature}\;{(}^{\circ }C)\\ T_{out} & =\mathrm{outdoor}\;\mathrm{temperature}\;{(}^{\circ }C)\\ M_{air} & =\mathrm{mass}\;\mathrm{of}\;\mathrm{air}\;\mathrm{inside}\;\mathrm{the}\;\mathrm{house}\;(\mathrm{kg}/s)\\ R_{eq} & =\mathrm{equivalent}\;\mathrm{thermal}\;\mathrm{resistance}\;\mathrm{of}\;\mathrm{the}\;\mathrm{house}\;(K/W)\\ S_{rad} & =\mathrm{solar}\;\mathrm{radiation}\;({W/m}^{2})\\ W_{area} & =\mathrm{total}\;\mathrm{window}\;\mathrm{area}\;(m^{2}) \end{array} \end{array}$$

**Figure 1:**
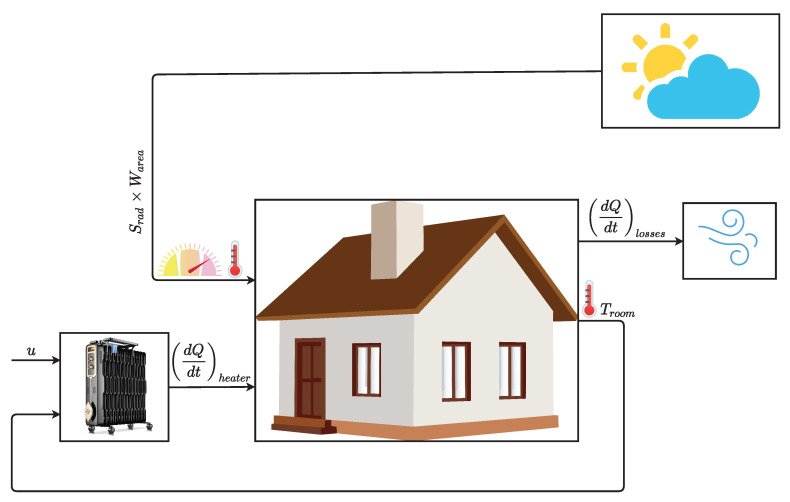
Illustration of the developed indoor environment model.

Figure 1 is a visual representation of the model adopted in this paper. There are two primary sources of heat (heater and solar radiation) and one source of losses. At the center is the building or room whose temperature should be controlled. This room acts as an integrator, as stated by the equation that computes the rate of change of its internal temperature $\left(\frac{\partial T_{room}}{\partial t}\right)$, meaning that the heat coming from the heating sources will be accumulated until reaching a thermal balance between input and output heat sources. Concerning the input sources, there is a standard electric heater, which also acts like an integrator, heating until the maximum average rating value ($T_{heater\_avg}$) with some time constant with respect to the control signal applied. There is also the contribution from the incident solar radiation ($S_{rad}\cdot W_{area}$). The temperature losses result from heat exchanges with the outdoor environment and depend on the building insulation type. In this study, a standard value for the equivalent thermal resistance is adopted, to make the results as generic and representative as possible [27].

## 4. Experimental Evaluation Setup

**Figure 2:**
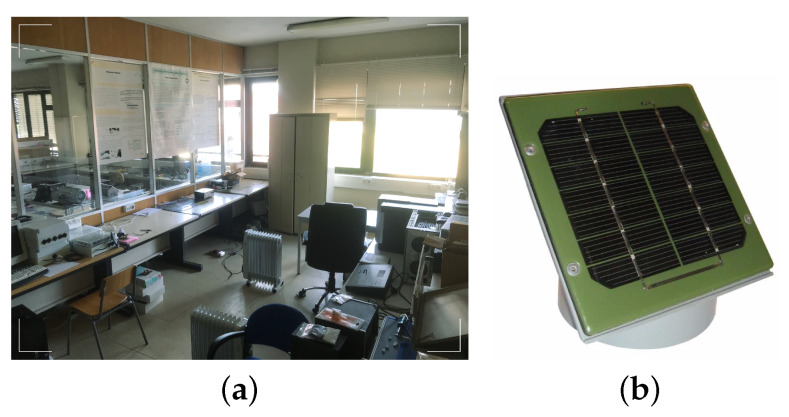
(**a**) Room used for the thermostatic control system evaluation; (**b**) photovoltaic panel used for the measurement of the solar radiation.

In order to validate the simulation results, a physical setup was deployed, composed of a distributed system integrating nodes to measure the necessary environment variables and actuate the heating system to control the indoor temperature. For this purpose, an Electronics and Control laboratory at the Department of Electronics, Telecommunications and Informatics of the University of Aveiro, shown in Figure 2a, was used. This laboratory has a single outside wall interfacing with the exterior, oriented towards the west and fully covered by glass windows, making it specially susceptible to the influence of direct solar radiation. The other walls, as well as the floor and the ceiling face other rooms and an internal corridor. Cooling is passive, resulting only from the heat transfer mechanisms to the adjacent spaces. Heating is carried out by three convective electric radiators evenly distributed in the room.

This is a challenging experimental setup since it has a high exposure to solar radiation and high and variable temperature losses, due to the intensive utilization of the adjacent corridors and divisions, which induce significant disturbances to the indoor temperature behavior.

**Figure 3:**
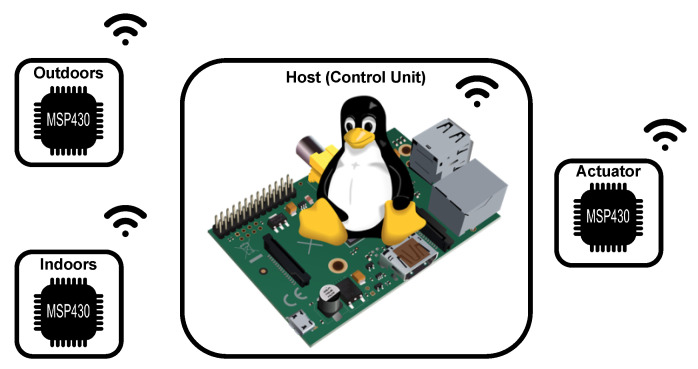
Illustrative diagram of the actuator system used during the real-word evaluation.

The main parameters of the environment and the implementation details are as follows:

- Room geometry:
  – Room length = 8 m;
  – Room width = 4 m;
  – Room height = 3 m;
  – Window area = 14 m${}^{2}$;
  – Wall area = 105 m${}^{2}$.
- Heating system technical specifications:
  – Maximum heating temperature = 60 ${}^{\circ }$C;
  – Heating power = 7500 W (3 × 2500 W).
- Actuator system specifications:
  – A Raspberry Pi Model 3B+ running a lightweight Linux distribution for running the Pole Placement algorithm was used. On the other hand, for measuring the input variables and actuating the electric heating system, a low-power MSP430 micro-controller from Texas Instruments coupled with an RFM69HCW radio from HopeRF for communicating with the Raspberry Pi was used. Figure 3 illustrates the global system architecture.

### 4.1. Measuring System Details

While ideally, it would be advantageous to have a full characterization of every single disturbance that may affect the control system, practical aspects such as cost and deployment complexity management tend to introduce compromises in the implementations. While temperature sensors with sufficient accuracy are relatively ubiquitous and inexpensive, measuring solar radiation typically requires the use of pyranometers, which are relatively expensive. This is probably one of the main reasons why Commercial Of-The-Shelf (COTS) temperature controllers do not use this variable in the decision process.

**Figure 4:**
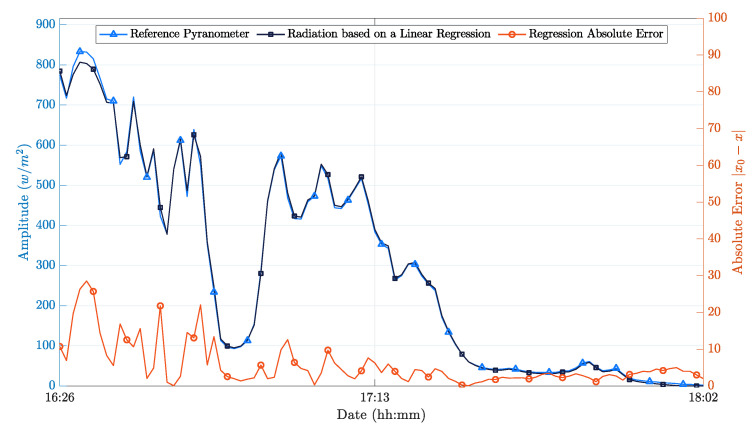
PV panel performance after calibration.

To circumvent this constraint, we propose the use of a PhotoVoltaic panel (PV) (Figure 2b), which converts the incident light into electricity using semiconducting materials that exhibit the photovoltaic effect [28]. This approach is capable of providing a close estimation of the solar radiation value while having a significantly smaller cost than pyranometers. To accomplish this, it is required to estimate the power delivered by the photovoltaic panel at a given instant, to know the panel dimensions and have a calibration value from a reference pyranometer, to obtain the panel efficiency constant (Equation (2)). Based on these elements, it becomes possible to obtain the required information regarding solar radiation with a small absolute error, as shown in Figure 4.

(2) $$P_{solar}=\frac{P}{\eta }$$

(3) $$Solar_{rad}=\frac{P_{solar}}{area}$$

Figure 4 shows an example of the measurements carried out, obtained in this case at the end of the winter season between 4:30 PM and 6:00 PM. We chose this time-frame to have a more significant variation of incident solar radiation, since the sunset at the end of the season is typically near 6:00 PM. It was a cloudy day, which further contributed to the appearance of significant solar intensity variations, allowing us to obtain more significant results. After calibration, it was possible to obtain an average absolute error of 5.7 W/m${}^{2}$ and an average relative error of 0.1%.

## 5. Adaptive Climatic Control System

The following subsection presents a short description of how the PP controller was synthesized.

### Pole-Placement

Pole-placement is a method used in feedback control systems that essentially consists of placing the closed loop poles of a plant in specific locations in the s-plan, in order to control the characteristics of the response of the resulting closed-loop system [29,30]. Typically, the controller parameters are obtained from a discrete-time model whose parameters are estimated online using the Recursive Least Squares (RLS) algorithm [31,32].

**Figure 5:**
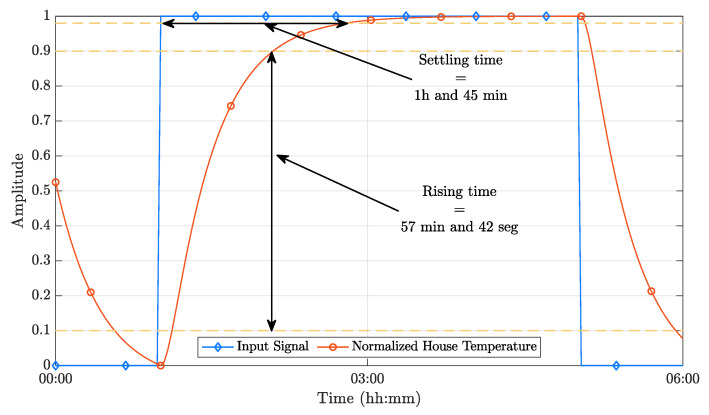
Step response of the dynamic model.

The scope of this work is assessing the potential gains of using the indoor and outdoor temperature, together with the incident solar radiation, in the RLS regression vector, instead of using only the indoor temperature. The discrete Auto-Regressive model with eXogenous inputs (ARX) of first-order dynamics has been demonstrated to be an effective technique to approximate the room temperature behavior when the heating system power level is adjusted, as shown in Section 6, Figure 5. Therefore, a PP control law based on the first-order model parameters was derived as follows [33,34]:

(4) $$u\left(k\right)=\left(\frac{b_{m}}{b}\right)\left(u_{c}\left(k\right)+a_{o}u_{c}\left(k-1\right)\right)-s_{0}y\left(k\right)-s_{1}y\left(k-1\right)+u\left(k-1\right)$$

(5) $$\begin{array}{c} \left\{\begin{array}{cc} s_{0}= & \frac{1-a+a_{m}+a_{o}}{b}\\ s_{1}= & \frac{a_{m}a_{o}+a}{b} \end{array}\right. \end{array}$$

where u(k) stands for the control signal applied to the system and $u_{c}\left(k\right)$ represents the reference signal that the controller follows. Additionally, and in accordance with the standard nomenclature, y(k) stands for the output signal (${}^{\circ }$C), and $a_{m}$ and $b_{m}$ are the discrete-time model coefficients related to the desired closed-loop model. Furthermore, the discrete-time model coefficients associated with the open-loop system model are represented by *a* and *b*, while $a_{o}$ is the discrete-time coefficient related to the observer polynomial [33].

## 6. Performance Evaluation: Simulation

This section presents the results obtained from the computer simulation of the implemented control system. These results were obtained using a combined simulation environment using MATLAB and the Simulink toolbox. The dynamic behavior of the house model was implemented using the Simulink toolbox, based on the continuous-time model derived from the thermodynamic equations presented in Section 3. The environment conditions were obtained from a dataset provided by [35], which makes the simulation close to a real implementation.

The following subsections present an analysis of the simulation results, starting with the ones obtained from the open-loop analysis of the thermodynamic model of the house (Section 6.1), followed by the assessment of the closed-loop performance of the controller both with and without the extra regressors in the RLS algorithm (Section 6.2).

### 6.1. Open-Loop Analysis

Before starting the test of the control algorithms, it is important to perform an open-loop analysis of the dynamic model in order to gather important details regarding the system behavior. By evaluating the step response analysis of the model, one can evaluate for example the accuracy of the thermodynamic laws implemented and obtain important parameters for tuning a significant number of controller synthesis approaches.

Figure 5 depicts the normalized outputs of the dynamic model. The required sampling time ($T_{s}$) can be readily obtained from the data presented in this figure. Considering that the ideal sampling time should include between 10 and 20 samples of the system’s rising time, it is possible to bound $T_{s}$ to $173\;s\leq T_{s}\leq 346\;s$; therefore, selecting $T_{s}=300\;s$ results in an adequate compromise. To be able to use a PP implementation, it is necessary to specify the desired closed-loop poles. Using a closed-loop system time constant with $\tau =5\;T_{s}$ results in a good compromise between performance and efficiency, as demonstrated in Appendix A. Therefore, a value of τ=1500s was selected.

### 6.2. Closed-Loop Analysis

The aim of this subsection is to evaluate the closed-loop performance of the different climate controller strategies. As mentioned in the Introduction, on-off is the most commonly used climate controller. The actuation of this controller is based on the instantaneous measurement of the indoor temperature. However, in the case of the PP controller, the regression vector comprises both the output and the input of the model, delayed by one sample (as required for modeling a first-order ARX model (Section 5). Additionally, in this specific PP implementation, the regression vector is enriched with the measurement of solar radiation and the outdoor temperature.

**Figure 6:**
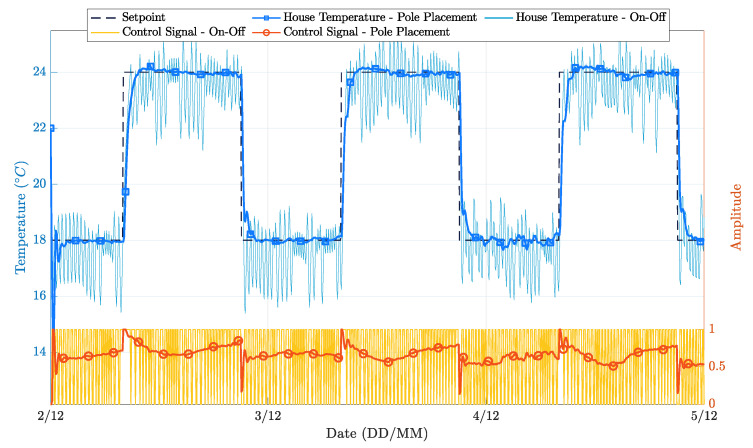
House temperature variation during the simulation time for PP and on-off controllers.

To better understand the implications of such approaches, the performance of the on-off and PP controllers are now presented. Figure 6 illustrates the evolution of the indoor temperature towards the chosen setpoint for the on-off and PP controllers. Since the on-off controller relies only on the current indoor temperature to decide if the heating system should be turned on or off, the indoor temperature presents an oscillation around the chosen setpoint. Even if human perception is hardly sensitive enough to perceive such a level of variation, this behavior causes a relevant waste of energy and a potential reduction of the actuation system lifetime. Moreover, there is little room for improvements on this controller, as the single parameter that can be tuned is the hysteresis of the controller around the setpoint region.

Figure 6 clearly shows that the PP controller outperforms the on-off controller, even during its initial learning period. Two different implementations of the PP controller are considered:

- Option 1: a first-order PP controller, where the RLS algorithm inputs are only the last indoor temperature sample and the last applied control signal;
- Option 2: a first-order PP controller, where the RLS algorithm inputs are extended to include, in addition to the last indoor temperature sample and the last applied control signal, external disturbances, namely the incident solar radiation and the outside temperature.

**Figure 7:**
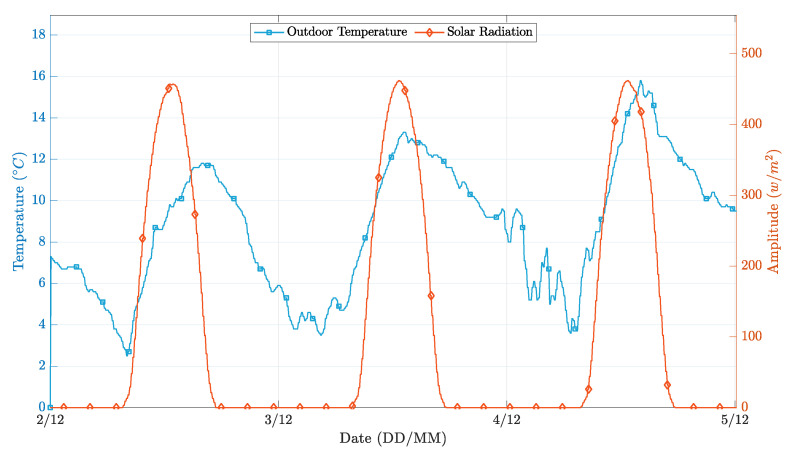
External environment conditions during the simulation time.

**Figure 8:**
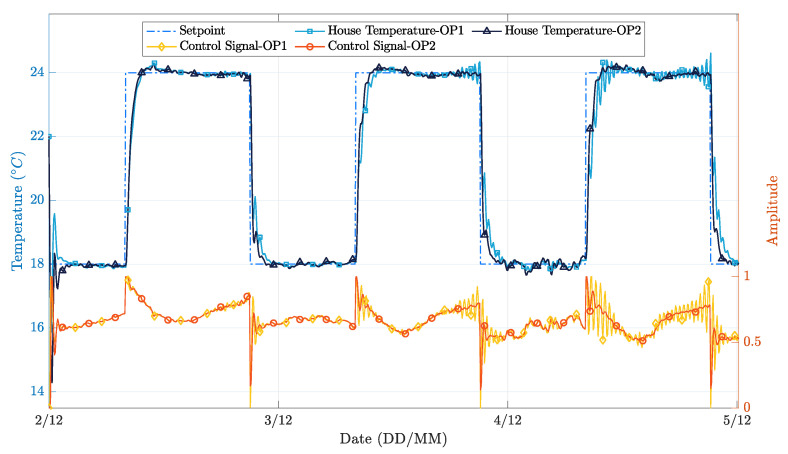
House temperature variation during the simulation time for two different implementations of the PP controller.

The controller’s performance was evaluated for a three-day time window, with clear sunny days and average temperatures typical of the winter season. Figure 7 depicts the behavior of the external environmental factors considered, and Figure 8 presents the different operation results.

In general, both PP realizations successfully followed the temperature setpoint while applying a control signal significantly smoother than the on-off realization. A more in-depth analysis, obtained by overlapping Figure 7 and Figure 8, reveals that the simpler implementation of the PP controller presents more significant oscillations in the instants where the external environment conditions change significantly. This phenomenon happens because the first-order model coefficients are wrongly estimated, as the estimator is not able to decouple the contribution of solar radiation and outdoor temperature from the contribution of the heating system, to the changes in the indoor environment temperature. Conversely, in the extended implementation of the PP controller, this situation does not occur because the regressors vector has more parameters, thus higher flexibility to decouple the influence of the solar radiation conditions from the heating actuation towards the indoor temperature variation.

Table 1 and Table 2 present quantitative results regarding the controllers’ performance. More specifically, Table 1 presents the Mean Squared Error (MSE) and Normalized Mean Squared Error (NMSE) for the three approaches. The NMSE formulation adopted in this paper is $NMSE=20\;\log_{10}\left(\sqrt{\sum_{i}{\left({\overset{\wedge }{x}}_{i}-x_{i}\right)}^{2}}/\sqrt{\sum_{i}\left(x_{i}^{2}\right)}\right)$. It is possible to observe that the PP controllers show an MSE reduction of 42% and 52% with respect to the on-off controller. Moreover, the extended PP controller exhibits a 17% MSE reduction with respect to the simpler one.

Another important aspect to consider is the variance of the control signal applied to the heating system, because it is well known that high variance signals may lead to faster degradation of heating system actuators (e.g., degradation of water valves in the case of radiant electric heaters) and also impact negatively the energy efficiency and lifetime of inverter class air conditioning systems. Table 2 shows the variance of the control signal. Again, the PP controllers clearly outperform the on-off controller, with gains of 94% and 96%. Additionally, the second implementation of the PP controller shows an improvement of 24% with respect to the simpler one.

In order to further evaluate the performance differences between the different controller implementations, the corresponding operational cost was also assessed. This metric is obtained by integrating the control signal applied during the experiments, which is proportional to the nominal power of the heating system. The obtained results show savings close to 14% when comparing the second implementation of the PP controller with the on-off controller and approximately 1% when comparing the second implementation of the PP controller with the first one. Once again, it is possible to conclude that on-off controllers perform worse than PP ones. On the other hand, according to this criterion, the performance difference between both PP controllers is marginal. It is, however, important to recall that the results respect only a three-day simulation and a set-up with convective electric radiators. For a longer evaluation period, the extended PP controller is better suited to deal with a broad diversity of external environmental conditions; thus, the small operation gains may become more relevant to the user. Moreover, with other heating sources, e.g., inverter air conditioners, that currently are quite common, the savings would be much more significant, both in terms of energy consumption and equipment lifetime. It is also important to point out that the additional implementation costs are not significant; thus, even a sustained 1% reduction in the heating energy consumption largely compensates the investment.

### 6.3. Discussion

The simulation results above allow us to take several conclusions regarding the relative merits of the control strategies considered in this paper. As expected, the on-off controller has the worst performance, as it relies solely on the instantaneous indoor temperature to decide if the heating system should be turned on or off. Despite not being significant enough to be perceived by human users, this behavior penalizes the energy consumption and, above all, presents undesirable consumption peaks and may result in a reduction of the actuation system lifetime.

In turn, both PP realizations outperform the on-off controller, being able to follow the temperature setpoint more closely and with smaller oscillations, while applying a control signal that is significantly smoother, with benefits in terms of peak energy utilization and preservation of the actuation system lifetime. Comparing now both PP realizations, the obtained results clearly show the performance gains that can be attained by including in the model the external variables. The simpler PP controller (PP-OP1) presents more significant oscillations, particularly when the external environment conditions change significantly, because the first-order model coefficients are wrongly estimated as a result of the estimator not being able to decouple the contribution of solar radiation and outdoor temperature from the contribution of the heating system to the evolution of the indoor environment temperature. The second PP controller (PP-OP2) takes these variables into account and so is more effective. The net result is a closer and more stable tracking of the setpoint, as well as a much more stable control signal.

From a quantitative point of view, it is possible to observe that the PP controllers exhibit an MSE reduction of 42% and 52%, respectively, when compared with the on-off controller, while the PP-OP2 controller shows a 17% MSE reduction compared with the PP-OP1 controller. In this regard, it is important to recall that the additional computational effort implied by PP-OP2 is minimal and that the obtention of the additional variables can be realized with low-cost hardware, as discussed in Section 4.1. Moreover, in many cases, buildings already have systems that acquire these variables, and so, in such cases, the implementation cost would be negligible. The more relevant challenge in this latter case is that there are no standards to share these data, so the firmware would have to be adapted for each specific case.

## 7. Performance Evaluation: Physical Installation

To verify if the results obtained by simulation are realistic, the controllers were deployed in the real-world scenario described in Section 4. As above, this section includes two subsections dedicated to the evaluation of the open-loop and closed-loop implementations.

### 7.1. Open-Loop Measurements

**Figure 9:**
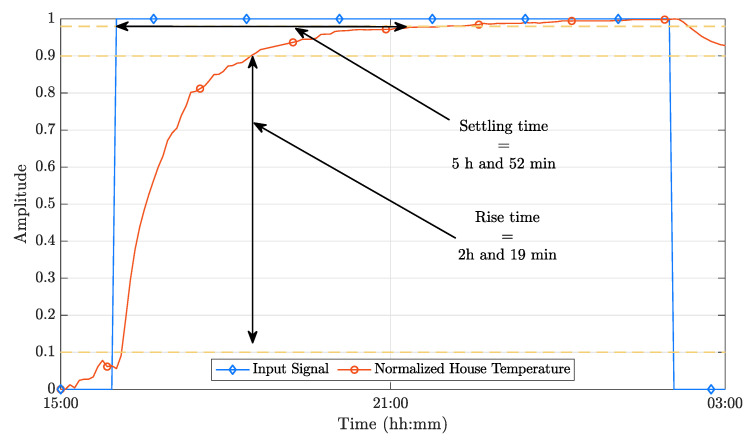
Step response of the system [5].

As previously mentioned, an open-loop analysis is fundamental to understand if the model is correct and also to get crucial parameters to synthesize the controllers. The step response obtained from the experimental data is shown in Figure 9. However, it is necessary to keep in mind that in real-world implementations, the external environment conditions often are not controllable. Thus, the step response is inherently affected by eventual variations of such conditions; therefore, its validity is restricted to certain boundaries.

Using the procedure presented in Section 6.1, it can be observed that $417\;s\leq T_{s}\leq 834\;s$. As before, an intermediate value for the sampling period, $T_{s}=600\;s$, was used, and the PP closed-loop time constant was set to $\tau =5\;T_{s}$.

### 7.2. Closed-Loop Assessment

This subsection presents the experimental results obtained with the on-off and PP-OP2 controllers. The results for PP-OP1 are not included because the test scenario is, as stated above, subject to significant perturbations (e.g., external climatic conditions, different number of users with different usage patterns). As such, the test conditions are not entirely replicable, making it difficult to extract meaningful conclusions, especially when the performance differences are not very pronounced. Moreover, the COVID-19 outbreak severely limited the number of experiments that were carried out, so it was not possible to obtain enough data for carrying out a proper comparison between PP-OP1 and PP-OP2. With this exception, the general procedure is the same as the one followed for the simulation scenario.

**Figure 10:**
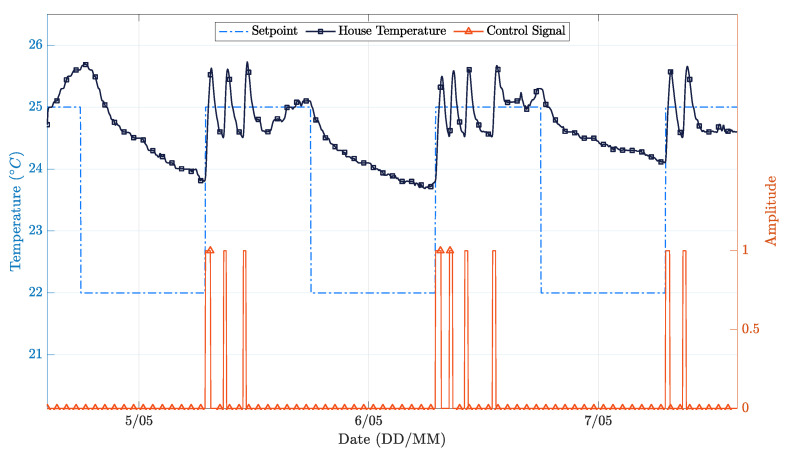
House temperature variation during test time on-off [5].

**Figure 11:**
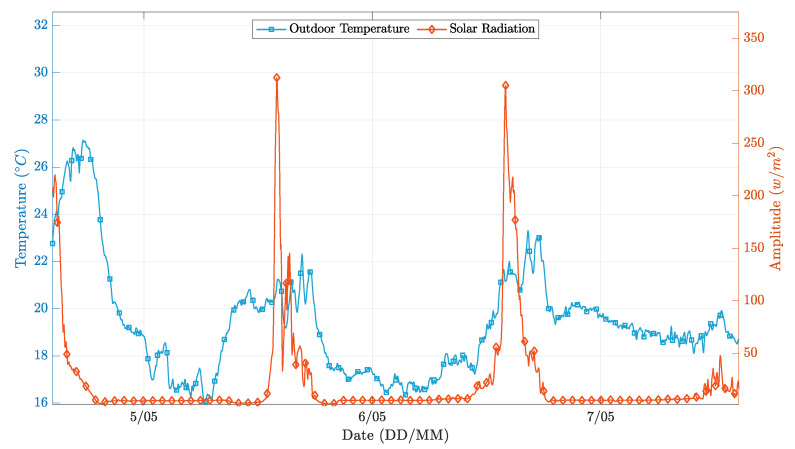
External environment conditions during test time on-off [5].

Figure 10 shows the overall performance of the on-off controller during a three-day test trial. It is possible to observe that the controller was able to follow the chosen temperature profile within a 2 ${}^{\circ }$C band. We also observe that the indoor temperature never dropped below 23.5 ${}^{\circ }$C because there was no assisted cooling appliance and the room losses were themselves insufficient for the indoor temperature to reach the setpoint (22 ${}^{\circ }$C). It is possible to better understand this by analyzing Figure 11, which illustrates the external environmental conditions during the three-day test trial. This figure highlights the impact of the incident solar radiation over the internal indoor temperature. By checking the control signal applied to the heating system in the first instants of the trial, one can see that this signal is zero, so the temperature should be dropping. Instead, the temperature is rising, revealing that there is a significant impact of those external environmental conditions over the indoor temperature. In short, this controller is straightforward to implement, but was revealed to be very ineffective, as expected.

**Figure 12:**
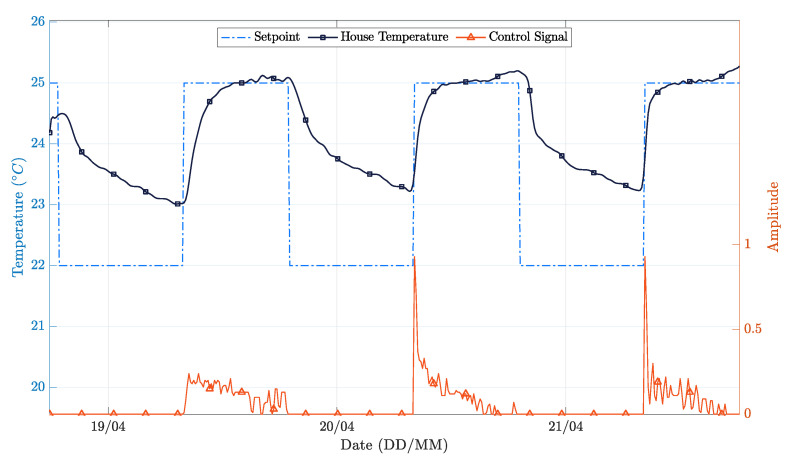
House temperature variation during test time PP [5].

**Figure 13:**
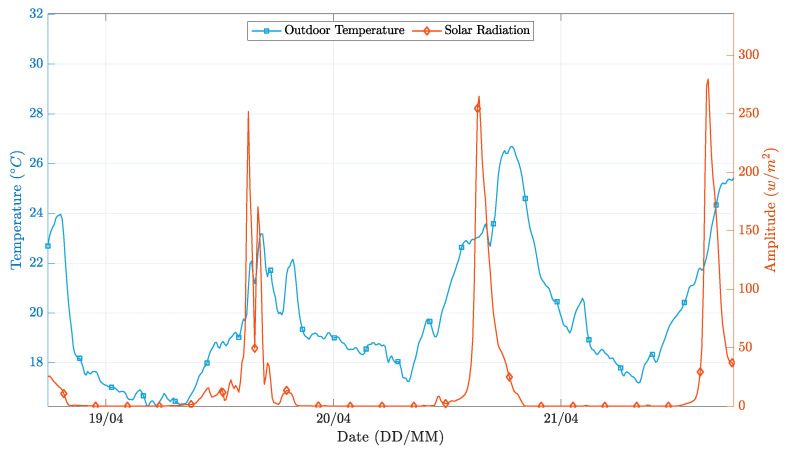
External environment conditions during test time PP [5].

Focusing now on the PP controller, Figure 12 shows the room temperature variation and the control signal applied to the heating system related to the setpoint. The setpoint is tracked with a good accuracy. Once again, one observes that the indoor temperature never drops below 23 ${}^{\circ }$C because there is no assisted cooling system installed and the evaluation period covered external climate conditions typical of summer days (Figure 13).

It can be observed that close to the end of days 20/4 and 21/4, there is a temperature peak. This behavior results from cooling being passive. As such, when the outside temperature reaches a given threshold, the indoor temperature rises, and the control system has no possible counter-action. On the other hand, a closer look at Figure 12 allows us to see that the settling time experiences a reduction over the test. This effect results from the online real-time RLS model identification method, which improves the model over time, thus allowing the controller to improve its performance.

The performance of the PP controller was not as good as expected. The main limitation is related to the lack of a cooling system. In fact, even knowing that the inside temperature will increase because of the incident solar radiation, the controller cannot avoid it. Eventually, a predictive controller could perform better, by turning off the heater sooner. In the simulation section, the benefit of measuring the external temperature and solar radiance was clearer because the exact same test environment was assured for all the controllers. While such evaluation conditions are not possible in real-world scenarios, the knowledge obtained from the simulation can be surely used to improve the stability of the discrete time model used for control synthesis in real-world implementations.

Table 3 and Table 4 summarize the quantitative results regarding the experimental evaluation. Keeping in mind that the evaluation conditions were not the same, the figures shown in Table 3 still confirm that the performance difference between the two controllers was significant. More specifically, the PP controller shows an MSE reduction of 45% and a 77% reduction in the control signal variance with respect to the on-off controller.

In realistic real-world scenarios with uncontrolled utilization and variable climatic conditions, as the one adopted in this study, computing a fair operational cost is a complex task. Nevertheless, to obtain an estimate for this parameter, the integral values of the variables were approximated using the trapezoidal method, and then, the integral of the control signal was multiplied by the integral of the incident solar radiation and by the integral of the outdoor temperature, in an attempt to include all the contributions coming from the different heating sources. With this method, it was estimated that the PP controller would show an improvement of 7% when compared to the on-off controller. It should be stressed that, as mentioned above, these results were obtained in different conditions (different days, different meteorological conditions, and different users and utilization patterns of the space), so they are merely indicative of potential behaviors and gains.

### 7.3. Discussion

Experiments were carried out only for the on-off and PP-OP2 controllers due to the difficulty of obtaining directly comparable data, as aspects such as the external climatic conditions, number of users, and usage patterns cannot be controlled in real scenarios. Moreover, the COVID-19 outbreak constrained the number of experiments that could be carried out.

Qualitatively, the practical results are in accordance with the simulation ones. The on-off controller is reveals to be highly sensitive to the incident solar radiation, exhibiting abrupt and significantly larger temperature oscillations than the PP-OP2 controller. Moreover, the control signal is much more unstable, having several commutations during some periods. Conversely, the temperature and control signal generated by the PP-OP2 controller are much smother. The temperature closely follows the setpoint, and the actuation peaks are sparse. As mentioned above, this behavior has benefits both in terms of energy management and in the actuator lifetime. Nevertheless, the performance of the PP controller was not as good as expected, mainly as a result of the use of thermal radiators that lack cooling capabilities. As such, for this kind of setup, predictive controllers may perform better, but at the expense of higher complexity.

From the quantitative point of view, it is possible to see that the on-off controller was only able to keep the temperature within a 2 ${}^{\circ }$C band, while the PP-OP2 controller was able to keep it within an interval of three tenths of a degree. Moreover, in the former case, the control signal had many abrupt oscillations, while in the latter one, it reached the maximum amplitude only occasionally, being in general below 20% of the maximum amplitude. Moreover, the PP controller showed an MSE reduction of 45% and a 77% reduction in the control signal variance with respect to the on-off controller. The operational cost was also estimated, showing an improvement of around 7% with respect to the on-off controller. As mentioned above, these results respect specific measurements taken over different days and conditions, so they must be regraded with caution. Nevertheless, they provide strong indications that the use of PP combined with external variables is effective when compared with on-off controllers. Moreover, this kind of controller can be realized with a minimum additional cost, unlike other approaches referred to in the state-of-the-art, so these potential gains largely compensate the additional complexity.

## 8. Conclusions and Future Work

This paper evaluates the potential benefits of using external environmental conditions, such as solar radiation and external temperature, in the design of advanced indoor climate control systems.

The proposed controller consists of a first-order PP controller, where the RLS algorithm inputs include the last indoor temperature sample and the last applied control signal, as well as external disturbances, namely the incident solar radiation and the outside temperature. The system was assessed against a simple on-off controller and an elemental PP controller, where the RLS algorithm uses only the last indoor temperature sample and the last applied control signal.

The simulation results show that PP controllers clearly outperform on-off controllers, with a reduction in excess of 33% in the error MSE and in excess of 90% in the control signal variance. Regarding these same parameters, the extended PP controller shows gains of 17% and 40%, respectively, with respect to the simpler PP controller. These figures have a significant impact on the performance, efficiency, and lifetime of equipment. For the specific scenario under test, the energy savings from the PP controller with respect to the on-off controller are close to 14%, with a marginal improvement of the improved PP controller with respect to the simpler one (≈1%). However, it is important to notice that the additional hardware required to deploy the extended PP controller is minimal and inexpensive and that for other types of heating systems, such as inverter air conditioning, such savings should be much more significant.

As concerns the algorithm implementation, the non-linear nature of the process was overcome by the discrete-time model identification procedure, which inherently updates a locally linear process model, which can be further used to obtain the closed loop controller gain parameters. Such a control synthesis approach enabled the use of advanced linear control strategies, elicited as a closed-form mathematical problem, the solution of which is implementable even in resource constrained systems.

The paper also includes experimental results obtained in a real-world setup, deployed in a laboratory room subject to normal utilization. The test conditions were naturally subject to significant variations among test runs, and the number of experiments was limited. Despite these constraints, the obtained results clearly confirm the significant performance differences between PP and on-off controllers. The practical realization of the system also allowed us to confirm that the system cost increase is modest; thus, the adoption of a more advanced controller is clearly advantageous.

As future work, the performance of other controller algorithms, namely the ones with predictive features, capable of covering longer time windows in advance so that weather forecasts could influence the control strategy, will be evaluated. While this approach allows higher performance levels, due to the use of longer term data, the integration of variables having a distinct nature, such as instantaneous temperature and solar radiation, with forecasts is challenging, particularly when facing highly dynamic scenarios, such as buildings subject to variable utilization patterns. Moreover, the system performance will also be evaluated with other types of actuators. The objective here is to investigate how well the learning process of the controller performs in the face of real-life actuator types that have heterogeneous characteristics, e.g., radiant floors, which only have heating capabilities and exhibit a slow response, and air-conditioning devices, which can heat and cool the indoor environment and have an almost instantaneous response. This a practical, but highly relevant aspect that needs to be thoroughly studied, in order to find how generic the approach is and what kinds of benefits are obtained in each case.

## Figures and Tables

**Table 1:** MSE and Normalized MSE (NMSE).

	MSE	NMSE
On-Off	1.6013	−24.59
PP-OP1	0.9277	−26.96
PP-OP2	0.7698	−27.77

**Table 2:** Control signal variance ($\sigma^{2}$).

	$\sigma^{2}$
On-Off	0.2276
PP-OP1	0.0129
PP-OP2	0.0098

**Table 3:** MSE and Normalized MSE (NMSE).

	MSE	NMSE
On-Off	3.1925	−22.35
PP	1.7507	−24.96

**Table 4:** Control signal variance ($\sigma^{2}$).

	$\sigma^{2}$
On-Off	0.0475
PP	0.0109

## Abbreviations

The following abbreviations are used in this manuscript:

ARX	Auto-Regressive model with eXogenous inputs
COTS	Commercial Of-The-Shelf)
HVAC	Heating, Ventilation, and Air Conditioning
MPC	Model Predictive Control
MSE	Mean Squared Error
NMSE	Normalized Mean Squared Error
PID	Proportional-Integral-Derivative
PP	Pole-Placement
PV	PhotoVoltaic
RLS	Recursive Least Squares
SHaB	Smart Homes and Buildings
T${}_{s}$	Sampling Time

## Appendix A

### Rule of Thumb for Choosing the Closed-Loop Time Constant for First Order Models

From [36] we got Equations (A1)–(A3).

(A1) $$\frac{T_{rise}}{20}\leq T_{sampling}\leq \frac{T_{rise}}{10}$$

(A2) $$T_{rise}=2.2\tau$$

(A3) $$T_{settling}=4\tau$$

As demonstrated by Equation (A4), choosing $\tau =5\times T_{s}$ compromises the closed-loop time constant between $0.55\tau_{open-loop}\leq \tau_{closed-loop}\leq 1.1\tau_{open-loop}$, which in worst case scenario will make the closed-loop time constant a copy of the open-loop time constant, or in the best scenario two times faster then the open-loop time constant.

(A4) $$\begin{array}{cc} \; & \frac{T_{rise_{open-loop}}}{20}\leq T_{sampling}\leq \frac{T_{rise_{open-loop}}}{10}\\ \Leftrightarrow & \frac{T_{rise_{open-loop}}}{20}\leq \frac{\tau_{closed-loop}}{5}\leq \frac{T_{rise_{open-loop}}}{10}\\ \Leftrightarrow & \frac{5\times 2.2\tau_{open-loop}}{20}\leq \tau_{closed-loop}\leq \frac{5\times 2.2\tau_{open-loop}}{10}\\ \Leftrightarrow & 0.55\tau_{open-loop}\leq \tau_{closed-loop}\leq 1.1\tau_{open-loop} \end{array}$$
